# Pathway mapping of leukocyte transcriptome in influenza patients reveals distinct pathogenic mechanisms associated with progression to severe infection

**DOI:** 10.1186/s12920-020-0672-7

**Published:** 2020-02-17

**Authors:** Yoann Zerbib, Emily K. Jenkins, Maryam Shojaei, Adrienne F. A. Meyers, John Ho, T. Blake Ball, Yoav Keynan, Amarnath Pisipati, Aseem Kumar, Anand Kumar, Marek Nalos, Benjamin M. Tang, Klaus Schughart, Anthony McLean, on behalf of the Nepean Genomic Research Group

**Affiliations:** 1 Department of medical Intensive Care, Amiens University Hospital, Amiens, France; 2 Department of Intensive Care Medicine, Nepean Hospital, Sydney, Australia; 3 Centre for immunology and allergy research, the Westmead Institute for Medical Research, Sydney, Australia; 4 National HIV and Retrovirology Laboratories, JC Wilt infectious disease research centre, Public health agency of Canada, Winnipeg, Canada; 5 Department of medical microbiology and infectious diseases, University of Manitoba, Winnipeg, Canada; 6 Department of internal medicine, medical microbiology and community health sciences, University of Manitoba, Winnipeg, Canada; 7 Department of chemistry and chemical biology, Harvard University, Cambridge, USA; 8 Department of chemistry and biochemistry, Laurentian University, Sudbury, Canada; 9 Section of critical care medicine and section of infectious diseases, department of medicine, medical microbiology and pharmacology, University of Manitoba, Winnipeg, Canada; 10 Department of Infection Genetics, Helmholtz Centre for Infection Research, Braunschweig, Germany; 11 University of Veterinary Medicine Hannover, Hannover, Germany; 12 Department of Microbiology, Immunology and Biochemistry, University of Tennessee Health Science Center, Memphis, Tennessee Germany

**Keywords:** Influenza, Transcriptome, Neutrophils, Neutrophil extracellular trap

## Abstract

**Background:**

Influenza infections produce a spectrum of disease severity, ranging from a mild respiratory illness to respiratory failure and death. The host-response pathways associated with the progression to severe influenza disease are not well understood.

**Methods:**

To gain insight into the disease mechanisms associated with progression to severe infection, we analyzed the leukocyte transcriptome in severe and moderate influenza patients and healthy control subjects. Pathway analysis on differentially expressed genes was performed using a topology-based pathway analysis tool that takes into account the interaction between multiple cellular pathways. The pathway profiles between moderate and severe influenza were then compared to delineate the biological mechanisms underpinning the progression from moderate to severe influenza.

**Results:**

107 patients (44 severe and 63 moderate influenza patients) and 52 healthy control subjects were included in the study. Severe influenza was associated with upregulation in several neutrophil-related pathways, including pathways involved in neutrophil differentiation, migration, degranulation and neutrophil extracellular trap (NET) formation. The degree of upregulation in neutrophil-related pathways were significantly higher in severely infected patients compared to moderately infected patients. Severe influenza was also associated with downregulation in immune response pathways, including pathways involved in antigen presentation such as CD4+ T-cell co-stimulation, CD8+ T cell and Natural Killer (NK) cells effector functions. Apoptosis pathways were also downregulated in severe influenza patients compare to moderate and healthy controls.

**Conclusions:**

These findings showed that there are changes in gene expression profile that may highlight distinct pathogenic mechanisms associated with progression from moderate to severe influenza infection.

## Background

Infection with influenza virus is a significant cause of morbidity and mortality worldwide and is seen as one of the major global health threats of our time [1]. Influenza can cause a mild respiratory illness but can also lead to respiratory failure and death [2, 3]. Virulence factors (of different virus strains) contribute significantly to this variability in infection severity [4, 5]. However, it is increasingly being recognized that host factors also play an important role in contributing to infection severity [6–10]. Recently, circulating leukocytes have been identified as the main host factors linked to infection severity, as revealed by transcriptomics studies [11–13]. Transcriptomics studies capture global gene-expression changes expressed by circulating leukocytes and findings from these studies showed that influenza host response displayed distinctively changes across the full range of mild, moderate and severe infection [12–16]. However, further advances are impeded by a major limitation in these studies. Transcriptomics studies provide gene-level analysis; they offer limited insight into the underlying biological pathways affected by influenza infection. A pathway-level analysis approach will be more informative since it provides a considerably greater amount of biologically relevant information and thus will allow a better understanding of the pathogenic mechanisms linked to disease progression.

In this study, we aimed to gain insight into influenza pathogenesis on a pathway level. We used a topology-based pathway analysis technique to study the leukocytes-mediated host response of patients with influenza infection. Topology-based techniques have the advantage over other analytical techniques in that they incorporate the interactions of different genes and proteins, potentially revealing far more biologically relevant information than standard “gene-set” pathway analysis techniques. A recent study showed that topology-based pathway analysis can better model the biological phenomena and more realistically reflect the host response [17]. Here, we applied topology-based pathway analysis on blood transcriptomics data obtained from patients with moderate and severe influenza infection. Our objective was to delineate, from a pathway perspective, the host response mechanisms associated with disease progression from moderate to severe influenza infection.

## Methods

### Setting and patients

We performed a multicentre prospective study to recruit adult patients (> 18 years) fulfilling the World Health Organization criteria of influenza-like illness (fever of 38 °C or higher, cough and illness onset within the last 10 days). Only patients with a positive test for influenza virus were included. Severe influenza illness was defined as a severe influenza pneumonitis that requires mechanical ventilation. Moderate influenza illness was defined as a significant symptomatic disease (resulting consultation to an emergency department) but did not require mechanical ventilation support. Two physicians independently assigned the patients to groups (moderate vs. severity), based on the following criteria (1) whether mechanical ventilation was used, (2) that influenza virus was confirmed on PCR of airway samples, (3), that the clinical features are consistent with influenza illness. Patients infected with other viruses than influenza were excluded from the study. Healthy control subjects were also included. Study protocol was approved by the institutional review board of each participating institution. Informed written consents were obtained from all study participants.

### Data collection

Demographic data included age, gender, comorbidities including chronic respiratory disease and standard laboratory test were obtained. Moreover, airway sample (nasopharyngeal swab, throat sample) and 2.5 ml peripheral blood sample in PAXgene tubes were collected. For those admitted in intensive care unit (ICU) and were under mechanical ventilation, additional respiratory samples were obtained. Virus testing consisted of nucleic acid PCR in order to detect influenza A, influenza B, respiratory syncytial virus, rhinovirus, parainfluenza virus and metapneumovirus.

### RNA extraction, normalisation and microarray analysis

In each sample, whole blood RNA was extracted from PAXgene tubes as per manufacturer’s protocol (QIAGEN PreAnalytiX – Blood RNA version 2; 2015). After checking RNA integrity on Bioanalyzer (Agilent Technologies; Waldbronn, Germany), 100 ng of total RNA was applied for Cy3-labelling reaction using the one color Quick Amp labelling protocol (Agilent Technologies; Waldbronn, Germany). Labelled cRNA was hybridized to Agilent 8x60k Human V3 (Design ID: 072363) microarrays for 16 h at 68 °C and scanned using the Agilent DNA Microarray Scanner. Results were then analysed using the R software package (version 3.1.3). Pre-processing steps included background correction, adding an offset of 50, quantile normalization and annotation using the limma package and Agi4x44PreProcess packages. Multi-group comparisons and identification of differentially expressed probe sets were performed using limma with Benjamini and Hochberg correction for multiple testing. Differentially expressed probesets (some genes are represented by several probesets) were identified based on an adjusted *p*-value of < 0.05 and exhibiting more than a two-fold difference in expression levels ([log^2^] > 1). Full dataset of the expression data is available at the National Centre for Biotechnology Information Gene Expression Omnibus (GEO accession number GSE101702).

### Statistical analysis

Continuous variables were expressed as mean and standard deviation (SD) and were compared by using Mann-Whitney U test. Categorical variables were expressed as numbers and percentages and were compared by the chi-square test or the Fisher exact test as appropriate.

### Pathway analysis

**Fig. 1:**
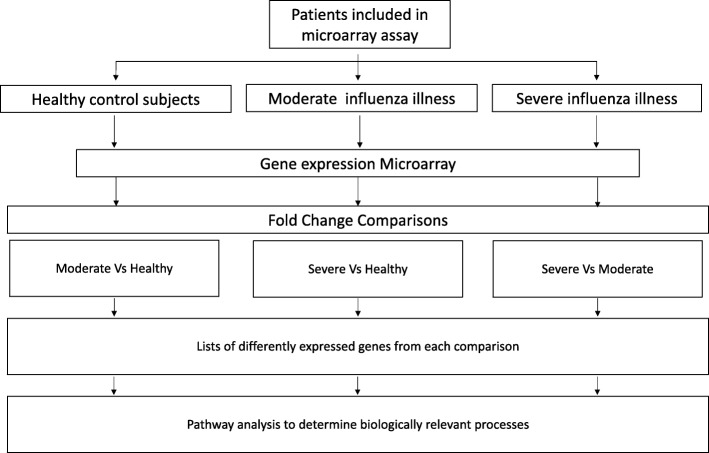
Flow chart and study scheme. Flow chart shows study design and analysis workflow

Three lists of differentially expressed genes were generated by comparing three different phenotypes (Fig. 1):

I. Moderate influenza vs. healthy controls
II. Severe influenza vs. healthy controls
III. Severe influenza vs. moderate influenza

Differentially expressed (DE) probesets between these comparison groups were identified based on an adjusted *p*-value of < 0.05 and exhibiting more than a two-fold difference in expression levels ([log^2^] > 1). Changes in expression levels were presented as fold changes for probesets of a given gene.

For pathway analysis, probeset lists were collapsed to gene lists (some genes are represented by multiple probesets that may be differentially expressed) and these three DE gene lists were used as input for the pathway analysis software, MetaCore. MetaCore is a topology-based analysis software (Clarivate Analytics, Philadelphia, U.S.A.) designed for functional analysis of transcriptomic data. Pathway analysis consists in examining the intersection between our lists of differentially expressed genes and prebuilt canonical pathways. A first statistical analysis is performed that take into account the number of genes differentially expressed in each comparison, the number of genes that intersect the prebuilt maps and the number of genes in the database. A False Discovery Rate (FDR) adjustment is apply for multiple testing. An FDR of 5% was used as the cut-off to determine whether a pathway was statistically overrepresented in the gene list. Adjusted *p*-value are expressed in -log(p-value) and ranked by statistical significance. Finally, these statistically overrepresented pathways were re-organized under broad biological themes (e.g. “Interferon response”, “Neutrophils”, “Immune response” and “Cell cycle”) [17–19].

## Results

### Clinical data

One hundred seven patients were recruited with laboratory-confirmed influenza infection (either nasopharyngeal swab or bronchoalveolar lavage (BAL)). There was no viral coinfection observed in the cohort. Microarray analysis was performed for all 107 samples. The moderate group and the severe group, as defined by clinical criteria (see Methods), consisted of 63 and 44 patients respectively. Patient assignment matches perfectly between the 2 experts. A group of 52 healthy control subjects without any medical illnesses had also been enrolled (Fig. 1). Baseline characteristics are described in Table 1. Age, gender, cell counts did not differ significantly between the groups. Eighty five percent (*n* = 91) of the patients had at least one comorbidity and the proportion was not significantly different between moderate and severe cases (*n* = 51 (81%), *n* = 40 (91%), *p* value: 0.18). In the moderate group, 45 patients (71%) required hospitalization and 7 (11%) required ICU admission. Severity disease was associated with a longer length of stay, (1.4 days vs 26 days; p value< 0,0001). The hospital mortality rate in the severe cases of influenza pneumonitis was 20% (9/44 patients).

**Table 1:** Demographics and clinical characteristics of patients

	Healthy Controls *n* = 52	Moderate *n* = 63	Severe *n* = 44	*p* values*
Gender (males/females)	19/33	27/36	17/27	0.69
Age/years (mean (SD))	43.5 (14.5)	52.6 (19)	46.5 (16)	0.11
Duration of onset (d)		4	5.6	0.19
Comorbidities n (%)	No Comorbidity	51	40	0.18
Asthma		8 (13%)	9 (20%)	0.25
Chronic lung disease		7 (11%)	9 (20%)	0.12
Cancer/ on chemotherapy		5 (7.9%)	2 (4.5%)	0.57
Ischemic heart disease		12 (19%)	6 (14%)	0.45
Hypertension		11 (17%)	5 (11%)	0.31
Diabetes		8 (13%)	9 (20%)	0.25
Cell counts				
Total leukocytes		8.1 × 1000/mm^3^	9.6 × 1000/mm^3^	0.76
Neutrophils		7.0 × 1000/mm^3^	7.4 × 1000/mm^3^	0.76
Respiratory support				
Invasive mechanical ventilation		0 (0%)	42 (95%)	< 0.0001
Non-invasive support (CPAP)		0 (0%)	2 (5%)	< 0.0001
Outcomes				
Hospitalization	NA	45 (71%)	44 (100%)	< 0.0001
Admission to ICU	NA	7 (11%)	44 (100%)	< 0.0001
Length of hospital stay	NA	1.4 days	26 days	< 0.0001
Death	NA	0 (0%)	9 (20%)	< 0.0001

* *p* values are calculated by comparing moderate and severe groups using Mann-Whitney test for continuous variables or Chi-square test for categorical variables. ICU denotes intensive care unit. NA denotes not applicable

### Gene expression profile in severe influenza illness differs from moderate influenza illness

**Fig. 2:**
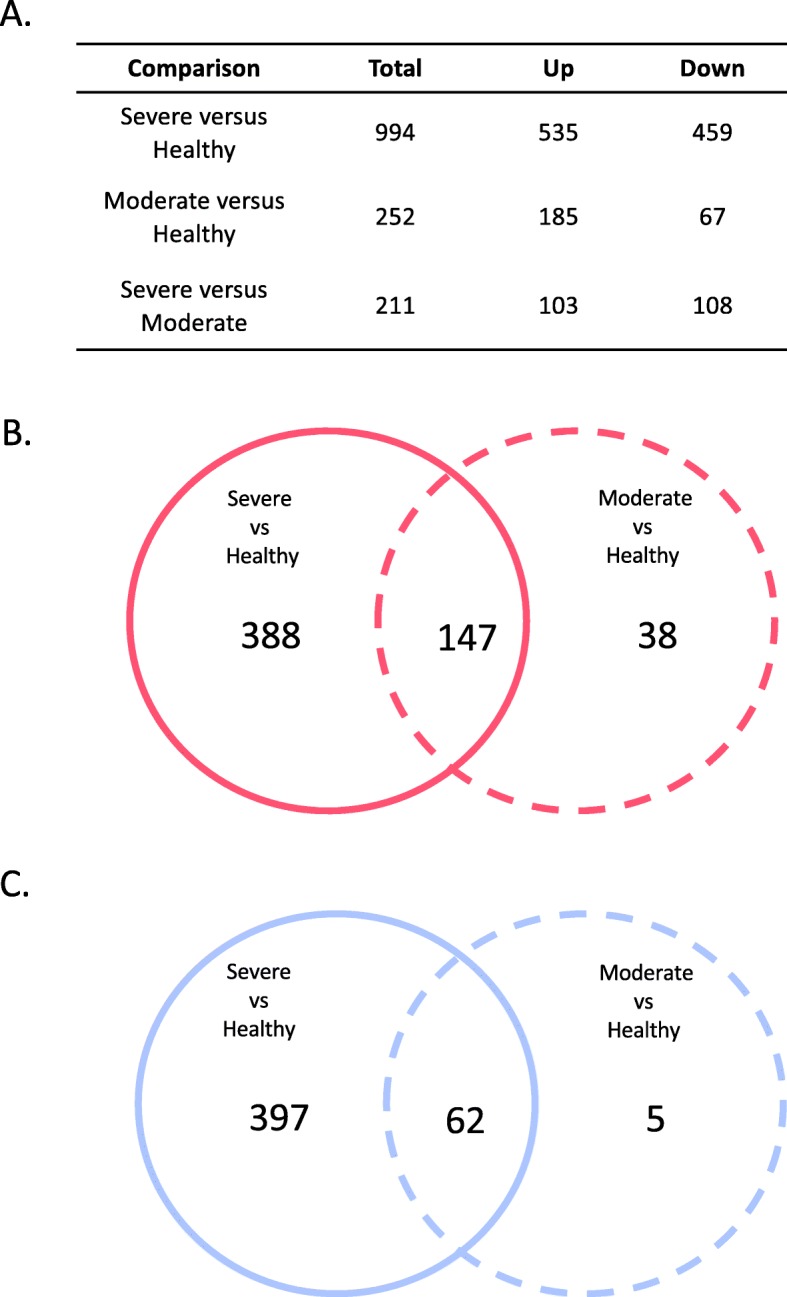
Differentially expressed genes in moderate and severe influenza. **a** Break down of statistically significant differentially expressed genes. The table showed the total number of differentially expressed genes in the three comparisons and the number of upregulated or downregulated genes. **b** Venn diagrams to indicate overlap of up-regulated genes. **c** Venn diagrams to indicate overlap of down-regulated genes. The Venn diagrams showed evidence that severe and moderate infection share common characteristics, but also have a distinctive gene expression profile

Influenza infection was associated with significant changes in gene expression. Compared to healthy control subjects, 994 transcripts from unique genes were found to be differentially expressed in severe influenza illness of which 535 were up-regulated and 459 down-regulated. Similarly, 252 transcripts from unique genes were differentially expressed in moderate influenza illness compared to healthy controls subjects of which 185 were up-regulated and 67 were down-regulated. Finally, the comparison between severe and moderate influenza illness revealed 211 transcripts (from unique genes) that were differentially expressed, of which 103 were up-regulated and 108 were down-regulated (Fig. 2a). Severe and moderate influenza illness share commonly expressed genes (147 up-regulated and 62 down-regulated). However, 388 genes were found to be up-regulated only in the severe influenza group and 38 genes only in the moderate influenza group. Conversely, 397 genes were found to be down-regulated only in the severe influenza group and 5 genes only in the moderate influenza group (Fig. 2b, c).

Unsupervised principal component analysis (PCA) was performed using normalized log_2_ gene-expression levels (Additional file 1: Fig. S1). Based on two principal components, the analysis showed a separation in the gene expression between severe influenza, moderate influenza and healthy control subjects. We note that gender didn’t seem to be associated with a separation in gene expression profile (Additional file 1: Fig. S1B).

**Fig. 3:**
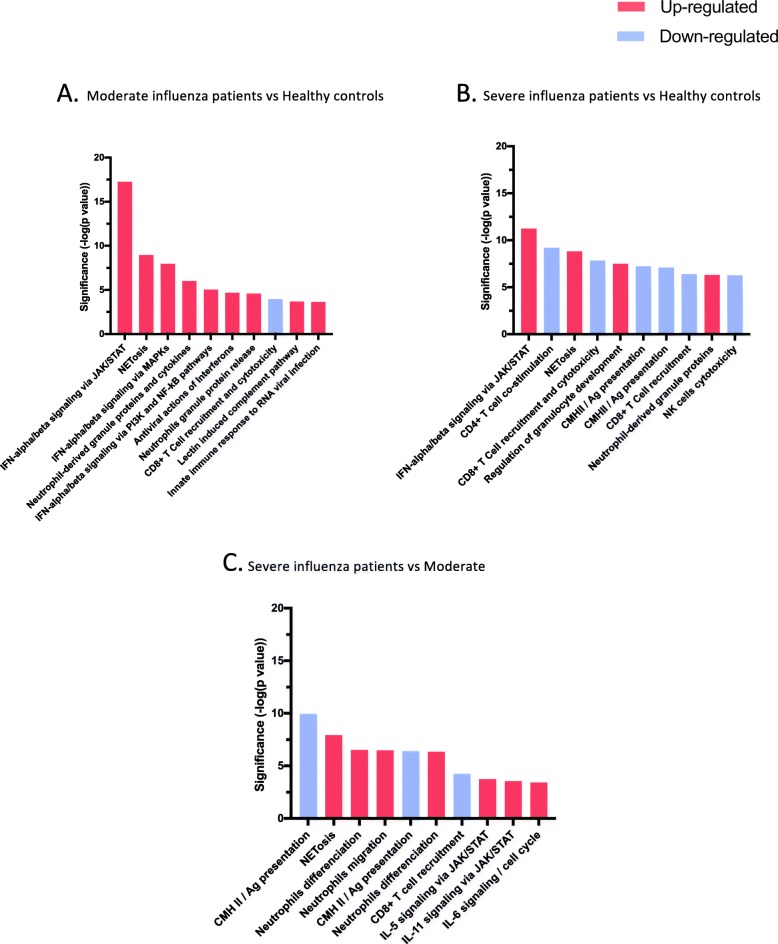
Top 10 pathways ranked by statistical significance. Top 10 pathways ranked by *p*-values (vertical bars) in three conditions. Vertical axis denotes statistical significance as measured by minus logarithm of *p*-values. Blue bars are downregulated pathways; red bars are upregulated pathways. **a** Moderate influenza patients compared to healthy controls. Upregulated pathways correspond to activation of interferon and neutrophil themes. Downregulated pathway corresponds to the immune response theme. **b** Severe influenza patients compared to healthy controls. Upregulated pathways correspond to activation of interferon and neutrophil themes. Downregulated pathway corresponds to the immune response theme. **c** Severe influenza patients compared to moderate influenza patients. Upregulated pathways correspond to activation of neutrophil and cell cycle (delayed apoptosis) themes. Downregulated pathway corresponds to the immune response theme

Taken together, these findings suggest that severe and moderate infection share common characteristics, but they also have a distinctive gene expression profiles, indicating severe and moderate infection might be associated with distinct host response. This was confirmed by subsequent pathway analysis, which revealed four biological themes (Interferon response, Neutrophils, Immune response and Cell cycle) of which three (Neutrophils, Immune response and Cell cycle) are differentially expressed in the severe group compare to the moderate group (Fig. 3, Additional file 1: Tables S1, S2, S3 and Additional files 2, 3 and 4).

### Interferon-related pathway genes

As expected, the most statistically significant pathways (in both moderate and severe groups) were those involved in antiviral response and interferon signalling. Notably, the interferon stimulated gene (ISG) *IFI27* was the most up-regulated transcript in almost all infected patients. Other transcripts from interferon-stimulated genes, with anti-viral functions, were also up-regulated in both moderate and severe groups compared to controls including *IFI44* (inhibits viral replication, *p* <  0.0001), *IFIT1* and *IFIT2* (inhibits viral mRNA translation initiation, *p* <  0.0001)*, MX1* (anti-influenza, *p* <  0.0001)*, OAS3* (degrades viral RNA, *p* <  0.0001). Conversely, we found only a small number of interferon stimulated genes (ISGs from the INTERFEROM database [20]) for the comparison between severe and moderate illness (Additional file 1: Figure S2). However, when we compared individual interferon-related genes, we observed a trend in downregulation of these genes in patients with severe illness (although these differences were statistically not significant) (Additional file 1: Figure S3). These findings showed that influenza infection up-regulates interferon-stimulated genes and other antiviral genes but the degree of up-regulation was not significantly different between severe and moderate illness.

### Neutrophil-related pathway genes

**Fig. 4:**
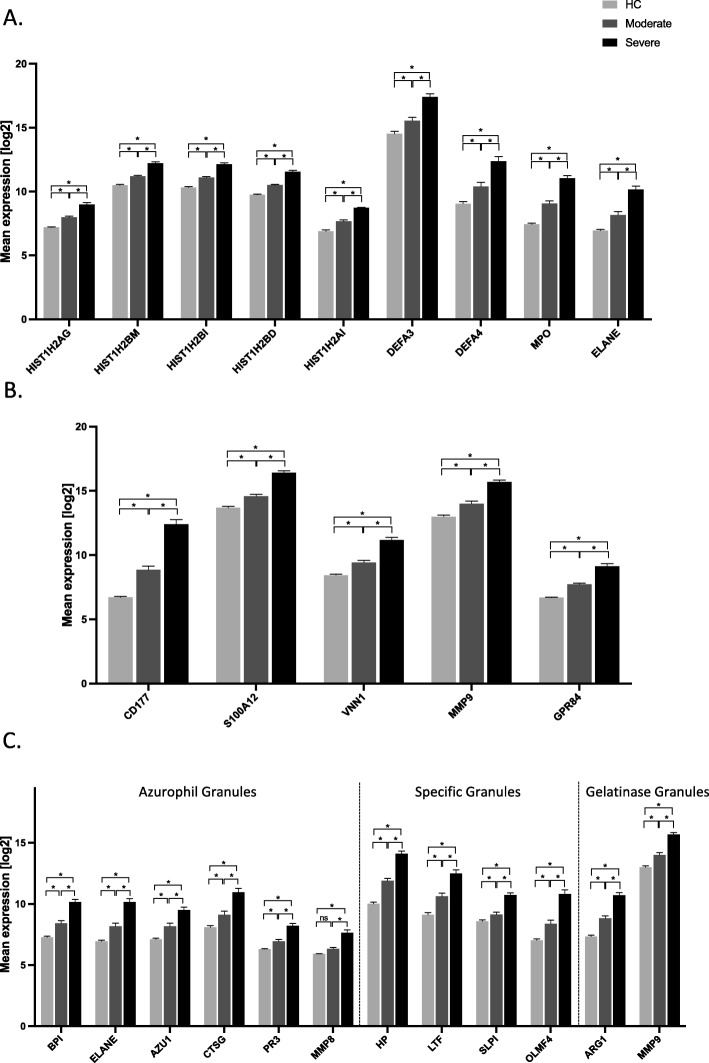
Histogram of neutrophil-related significant changes in gene expression between severe influenza, moderate influenza illness and healthy controls. Y-axis shows normalised log_2_ expression levels. * indicate *p* < 0.001, adjusted for multiple testing by Bonferroni method. ns denotes non-significant. HC denotes healthy control. **a** Genes encoding proteins involved in neutrophil extracellular trap formation. Expression differences are shown for (the strongest regulated) probesets of the individual gene. **b** Genes encoding proteins involved in neutrophil migration. Expression differences are shown for (the strongest regulated) probesets of the individual gene. **c** Genes encoding components of neutrophil granules. Expression differences are shown for (the strongest regulated) probesets of the individual gene. Neutrophils-related genes were upregulated in patients with severe influenza illness compared to moderate influenza illness and healthy control subjects

We also identified neutrophils-related pathways as the most strongly associated with severe disease. These pathways were involved in key neutrophil process including neutrophils differentiation, degranulation and neutrophil extracellular traps (NETs) formation (Fig. 4). Furthermore, transcripts from genes involved in driving neutrophil differentiation from myeloid cell lineages were significantly up-regulated in severe influenza. Among them, *RETN* (resistin), also known as *C/EBP-ε*, is a critical transcription factor and was highly up-regulated in severe influenza patients (*p* <  0.0001) [21, 22]. *RETN* induce myeloid/granulocyte-specific genes expression such as *PRTN3* (proteinase 3) *MPO* (myeloperoxidase), *LCN2* (lipocalin 2) and *LTF* (lactotransferrin), which promote cell differentiation into mature neutrophils. Other transcripts from genes encoding for specific neutrophils granules were also more highly expressed in severe patients (*MMP-9* (matrix metallopeptidase-9, *p* <  0.0001), *HP* (haptoglobin, *p* <  0.0001), *OLM4* (olfactomedin, *p* <  0.0001). We noted that *CD177* was the most abundant transcript in severe influenza illness compared to moderate illness and healthy control subjects (*p* <  0.0001). CD177 is a specific neutrophils protein that plays a role in neutrophils adhesion and transendothelial migration [23].

NETosis was also one of the most up-regulated pathway in severe influenza illness. Among transcripts from genes involved in NETs, all were significantly more expressed in severe influenza compared to moderate influenza (Fig. 3c). Furthermore, among the 30 most up-regulated transcripts in the severe group compared to the moderate group, 25 transcripts (83%) are from neutrophils related genes (Additional file 1: Table S4).

These findings suggest that, at the pathway level, neutrophil differentiation, activation, migration and finally NET formation are associated with a more severe disease in influenza patient.

### Immune response-related pathway genes

Compared to healthy control subjects, data from influenza patients showed a downregulation of the immune response pathways. Among the top 10 ranking pathways of biological significance in the moderate group, one pathway was associated with downregulation of CD8+ T cell recruitment and cytotoxicity. In the severe influenza group, there was a stronger effect of down-regulation in immune pathways; six pathways from the top 10 ranking pathways of biological significance were down-regulated (*p* <  0.0001). These pathways are involved in both innate and adaptive immune response: CD4+ T cell co-stimulation, MHC class II expression and antigen presentation, CD8+ cytotoxicity and NK cell cytotoxicity (Fig. 3).

**Fig. 5:**
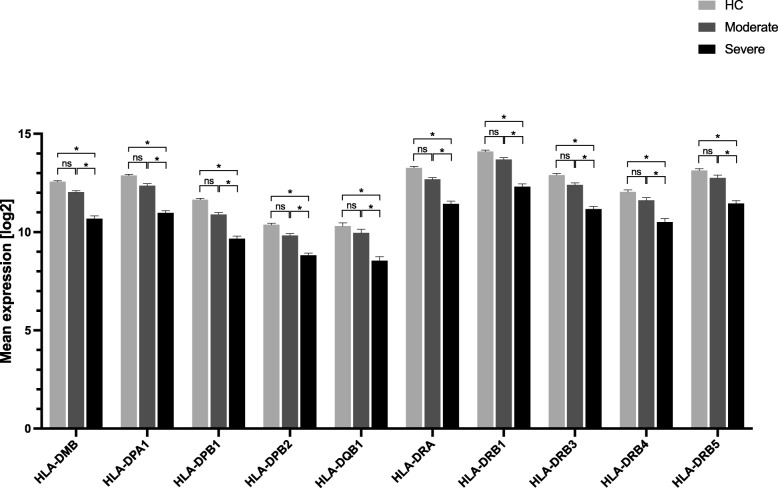
Histogram of MHC class II significant changes in gene expression between severe influenza compared to moderate influenza illness and healthy controls. Expression differences are shown for (the strongest regulated) probesets of the individual gene. Y-axis shows normalised log_2_ expression levels. * indicate *p* < 0.001, adjusted for multiple testing by Bonferroni method. ns denotes non-significant. HC denotes healthy control MHC class II were downregulated in patients with severe influenza illness compared to moderate influenza illness and healthy control subjects

**Fig. 6:**
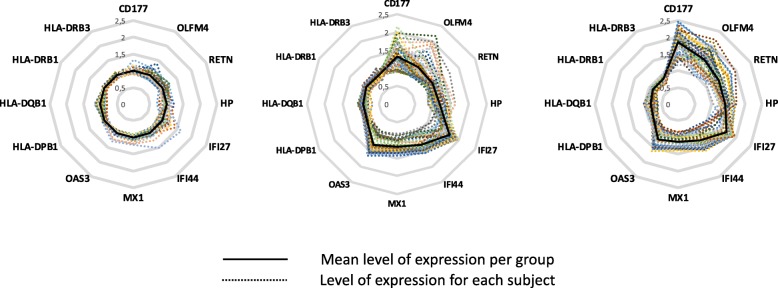
Radar chart: gene expression profile in moderate and severe influenza illness. Most representative differentially expressed probesets of the individual gene in the top three principal biological themes were computed in a radar chart. Level of expression was normalized to healthy controls. Expression differences are shown for (the strongest regulated) probesets of the individual gene. In moderate infection, the radar chart showed an upregulation of the interferon and neutrophil genes. In severe infection, besides an upregulation of the interferon and neutrophil genes, we observed a down regulation of the MHC class II genes

Comparing transcriptomes between severe and moderate influenza illness, we observed that the most down-regulated transcripts were from genes coding for Major histocompatibility complex (MHC) class II (*HLA-DRB5, HLA-DRA1, MHC class II alfa and beta chain, HLA-DRB1, HLA-DRB4, HLA-DRB3, HLA-DRB*) (p <  0.0001). Transcripts involved in the CD8+ T cell recruitment pathway also was found to be differentially expressed between moderate and severe illness but involved only a small number of transcripts (*CX3CR1* downregulation, *IL-18R1, ELANE, MMP-9* and *SLPI* upregulation) (Additional file 1: Table S3). These findings are summarised in Figs. 5 and 6.

### Cell cycle pathway genes

Cell cycle was also a distinct biological theme that we observed in severe patients. *BIRC5* (baculoviral IAP repeat containing 5) also known as *survivin* was up regulated in severe patients and is known to play a role in apoptosis inhibition (p <  0.0001). *CCNB2* (cyclin B2) was also up-regulated and encodes an essential component of the cell cycle regulatory machinery (*p* < 0.0001). Moreover, *CDKN1C* (Cyclin Dependant Kinase Inhibitor 1C) is a negative regulator of cell proliferation. We observed a downregulation of *CDKN1C* transcript in the severe influenza cases compared to moderate influenza cases (p < 0.0001). These findings suggest that cell proliferation and decrease in apoptosis could be associated with severity in influenza patients.

### External validation

We also performed a gene-based comparison with an external dataset (GEO 111368) [12]. This external dataset has a similar study design to ours, which included 109 adult patients with laboratory-confirmed influenza infection and 130 healthy participants. We found strong similarity in the upregulated genes between the two studies: 17 upregulated genes were shared between two studies (70%) (Although only 3 (15%) downregulated genes were shared between the two studies). Notably, the upregulated genes encode proteins involved in neutrophil functions and the downregulated genes encode proteins involved in the immune response. Among the most differentially expressed genes, neutrophil activation and reduced immune response were the dominant biological themes found in both dataset (Additional file 1: Table S5).

## Discussion

A large number of studies have focused on the immunological mechanism underpinning host responses against influenza infection. These studies have identified risk factors for progression to severe influenza infection including viral factors (subtype of influenza virus, viral load, mutation in viral genome) and host factors (genetic susceptibility, pre-existing medical conditions) [7–10]. Host factors play a protective role against influenza infection, but also may contribute to immunopathology, leading to tissue damage, organ failure and disease severity.

We herein report a study of systemic host factors in a large cohort of patients presenting with influenza illness and well characterized different level of severity. Pathway mapping of leukocyte transcriptome in influenza patients has revealed distinct pathogenic mechanisms associated with severe infection. These mechanisms were represented by four biological functional themes associated with influenza illness (Neutrophils, Immune Response, Cell cycle and Interferon response). Three of the biological themes (Neutrophils, Immune Response, Cell cycle) were more highly associated with severe infection. Of these three themes, the neutrophils theme was the most represented in severe illness. These results were consistent with an unsupervised analysis previously published by our team [13]. In that analysis, a weighted gene co-expression analysis (WGCNA) was performed to identify disease modules associated with infection severity. The results of that analysis are in keeping with the main findings of the current paper, which include (1) the neutrophil module displayed the highest increase in modular expression as influenza severity progress from moderate to severe form, (2), cell cycle module was upregulated, (3) immune response module revealed broad downregulation in gene expression of key genes involved in innate and adaptive immunity. Overall, the pathway analysis presented in this paper extends the previous WGCNA analysis by providing additional insights on the biological pathways associated with severe influenza infection.

The neutrophils theme consisted of several key pathogenic pathways, including neutrophils differentiation, neutrophils migration and degranulation and neutrophil extracellular traps formation. These findings are consistent with previous studies highlighting the complex dual role of neutrophils in influenza infection. Neutrophils are key cells in innate immune responses that can be protective during influenza infection [24, 25]. However, mice and human studies showed that lung damage can also be associated with neutrophils infiltration, matrix metalloproteinase 9 and myeloperoxidase activities [26–28]. Interestingly, alveolar damage was less severe in neutrophils depleted mice infected with influenza A H1N1 illustrating the dual roles of neutrophils (protective vs. harmful) in influenza pneumonitis [29]. NETs were also strongly induced in neutrophils from infected mice lungs and were responsible for an increase in endothelial damage. Moreover, it has been shown that NET’s formation is dependent on redox enzymes activation such as myeloperoxidase and superoxide dismutase and blocking these enzymes can reduce alveolar damages [30]. In line with these findings, extracellular histones, major components of NET’s, play an important role in causing lung injury in mice infected by influenza, most probably mediated by cytotoxicity and thrombus formation after platelet binding [31]. Taken together, these data support the possible role of neutrophils in severe influenza immunopathology and suggest that more studies are needed to explore whether neutrophil can be a potential target of host-directed therapy [12].

Interferon has been identified as a critical key host response against influenza infection. First, inborn errors on immunity in IRF7 and IRF9 have been identified as responsible for life threatening pulmonary influenza. These patients cell fail to amplify type I and III IFNs and to control viral replication [7, 8]. Second, previous study in severely infected patients revealed an increase of neutrophils transcripts while interferon-related transcripts were down-regulated [12]. Finally, impaired INF production is responsible for reduced immune responses in lungs leading to acute lung injury [8, 10]. We found that interferon genes were not differentially expressed between moderate and severe infection. The lack of a further up-regulated interferon response observed in the severe group is consistent with the hypothesis that a reduced interferon response may contribute to compromised host response. Therefore, it is possible that an insufficient interferon response may be linked with a deregulated neutrophil activation. Whether this is indeed true will require further mechanistic study (e.g. in animal model).

Neutrophilia is well known marker of bacterial infection. In this study, super-imposed bacterial infections may explain the upregulation of neutrophil pathway observed in severe infection. Therefore, additional studies are required to investigate if the neutrophil signature was associated with bacterial co-infection.

Finally, a previous in vitro study suggests that H1N1 influenza virus could replicate in and be released from neutrophils. It has also been shown that influenza virus itself can activate neutrophils and induce oxidative burst [32]. Whether these mechanisms could explain our observations is uncertain and will require additional studies in the future.

Our findings also suggested significant changes in the immune response at a transcriptomic level, including, impairment of NK cells cytotoxicity, CD4 T cells co-stimulation, T cell CD8 recruitment and MHC II antigen presentation. Effective antigen presentation is critical to develop a robust adaptive immune response to the virus, and without this response virus clearance cannot occur effectively. Effective antigen presentation requires the cooperative interaction between MHC II molecules, CD74 protein from the endoplasmic reticulum (ER) and T-cell-receptor (TCR)-CD3 complex. This interaction is followed by binding of the CD4 co-receptor to the MHC II complex and this binding is a critical step in initiating the signalling pathways leading to T-cell activation and differentiation. In the current study, we observed that many transcripts involved in these processes were down-regulated in patients with severe influenza illness compare to moderate patients and healthy control. These findings are consistent with previous reports, showing that NK cell cytotoxicity and specific CD8+ T-cell response are essential for protection against severe influenza disease [33, 34]. In line with these findings, it has been shown that memory cross reactive CD8+ T Cell may provide protection in case of infection. Thus, patients a lower count of pre-existing cross reactive CD8+ T cell could develop more severe illness [33, 34]. Interestingly, *ARG1* (arginase 1) was one of the most abundant transcripts found in severe influenza patients (Additional file 1: Fig. S5). ARG1 is known to be stored in granules of neutrophils. Once released and activated, ARG1 can degrade extra cellular arginine resulting in inhibition of T-cell proliferation [35].

CD8+ T-cell seems to play dual roles in influenza pathogenesis. If CD8+ cytotoxicity and the production of pro-inflammatory cytokines (INF-γ TNF-α) are critical for the efficiency of infection resolution in mice, they may also contribute to immunopathology and lung injury [36]. Our current study does not provide sufficient data to determine whether decreased CD8+ cytotoxicity could have contributed to immunopathology.

Apoptosis is also a well-known defence mechanism against viruses leading to the inhibition of the replication and the spread of virus into host [37]. Our data are consistent with previous work showing severe influenza infection results in the dysregulation of apoptosis in infected cells [38].

In summary, our transcriptomic data showed that, increase of neutrophils activity, decrease of antigen presentation and cytotoxicity (CD8+ T-cell and NK cells) are associated with severity while interferon pathway activation was common in all infected patients (Fig. 6).

Some limitations have to be acknowledged. An important limitation lies in the nature of the data we used. Our transcriptomic data requires further validation on both protein and functional levels. Also, it is important to keep in mind that our observations suggest biological and clinical association. However, establishing a causal link is not possible to do in humans but will require controlled animal experiments. Moreover, the immune response is a dynamic process and different pathways may be involved at different time points during an influenza infection. Also, patients arrive in hospital with different delay after onset. Although we found that the time elapsed since symptom did not statistically significantly impact on gene-expression levels, we cannot confidently exclude its potential confounding effect. Therefore, this issue should be clarified in future studies.

## Conclusion

This study provides evidence that influenza infection severity is associated with changes in gene expression profile. It allows hypothesis-generation suggesting that excessive neutrophil activation, impaired adaptive immune function and apoptosis may be important host factors in mediating the progression to severe influenza disease. Further studies are needed to confirm these findings and to explore host mechanisms in particular in neutrophil-related host response.

## Supplementary information

**Additional file 1: Figure S1.** Principal component analysis. **Figure S2.** Number of differentially expressed genes and proportion of interferon-stimulated gene for the three comparisons. **Figure S3.** Boxplots of Interferon-stimulated genes-expression. **Table S1.** Top 10 pathways ranked by statistical significance - moderate influenza patients compared to healthy controls. **Table S2.** Top 10 pathways ranked by statistical significance - severe influenza patients compare to healthy controls. **Table S3.** Top 10 pathways ranked by statistical significance - severe influenza patients compare to moderate influenza patients. **Table S4.** Top 30 differentially expressed genes (ranked by expression levels) between moderate and severe infections. **Table S5. a** Top 20 most upregulated genes in both datasets. **b** Top 20 most downregulated genes in both datasets.

**Additional file 2.** List of differentially expressed genes moderate vs healthy controls.

**Additional file 3.** List of differentially expressed genes severe vs healthy controls.

**Additional file 4.** List of differentially expressed genes severe vs moderate.

## Data Availability

Full dataset of the gene-expression data is available at the National Centre for Biotechnology Information Gene Expression Omnibus (GEO accession number GSE101702). Additional data can be obtained by contacting our data manager by email: sally.teoh@health.nsw.gov.au.

## Abbreviations

BAL: Bronchoalveolar Lavage
DE: Differentially Expressed
GEO: Gene Expression Omnibus
ICU: Intensive Care Unit
ISG: Interferon Stimulated Gene
MHC: Major Histocompatibility Complex
NET: Neutrophil Extracellular Trap
NK: Natural Killer
