# Supplementary material for: Pathway mapping of leukocyte transcriptome in influenza patients reveals distinct pathogenic mechanisms associated with progression to severe infection
Source: BMC Med Genomics. 2020 Feb 17;13:28. doi: 10.1186/s12920-020-0672-7 (PMC7027223; doi:10.1186/s12920-020-0672-7)
Supplement: Supplementary file 1 — Additional file 1: Figure S1. Principal component analysis. Figure S2. Number of differentially expressed genes and proportion of interferon-stimulated gene for the three comparisons. Figure S3. Boxplots of Interferon-stimulated genes-expression. Table S1. Top 10 pathways ranked by statistical significance - moderate influenza patients compared to healthy controls. Table S2. Top 10 pathways ranked by statistical significance - severe influenza patients compare to healthy controls. Table S3. Top 10 pathways ranked by statistical significance - severe influenza patients compare to moderate influenza patients. Table S4. Top 30 differentially expressed genes (ranked by expression levels) between moderate and severe infections. Table S5. a Top 20 most upregulated genes in both datasets. b Top 20 most downregulated genes in both datasets. [file 12920_2020_672_MOESM1_ESM.docx]

**ADDITIONAL FIGURES**

**Additional Figure 1 -**

**Additional Figure 2 -**

**Additional Figure 3 –**

**Additional Figure 1**

**A.**

**
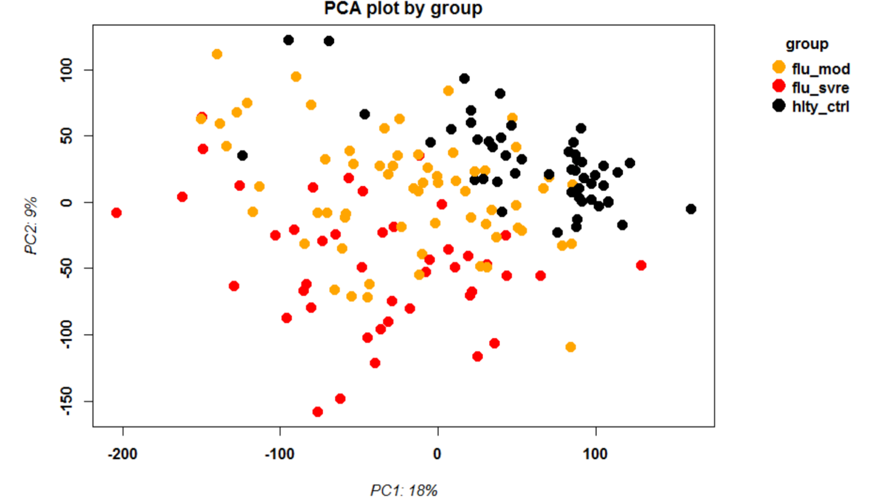
**

**B.**


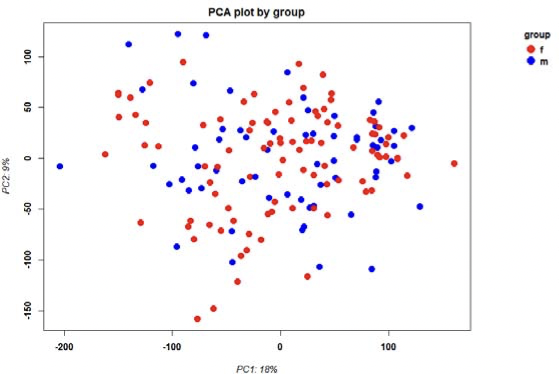


**Additional Figure 1: Principal component analysis.** Using normalized log_2_ gene-expression level, principal component analysis was performed. **A:** Based on two principal components, the three groups showed evidence of distinct gene-expression profile. **B:** Gender did not affect gene expression profile.

f denotes Female, m denotes Male

**Additional Figure 2**

**
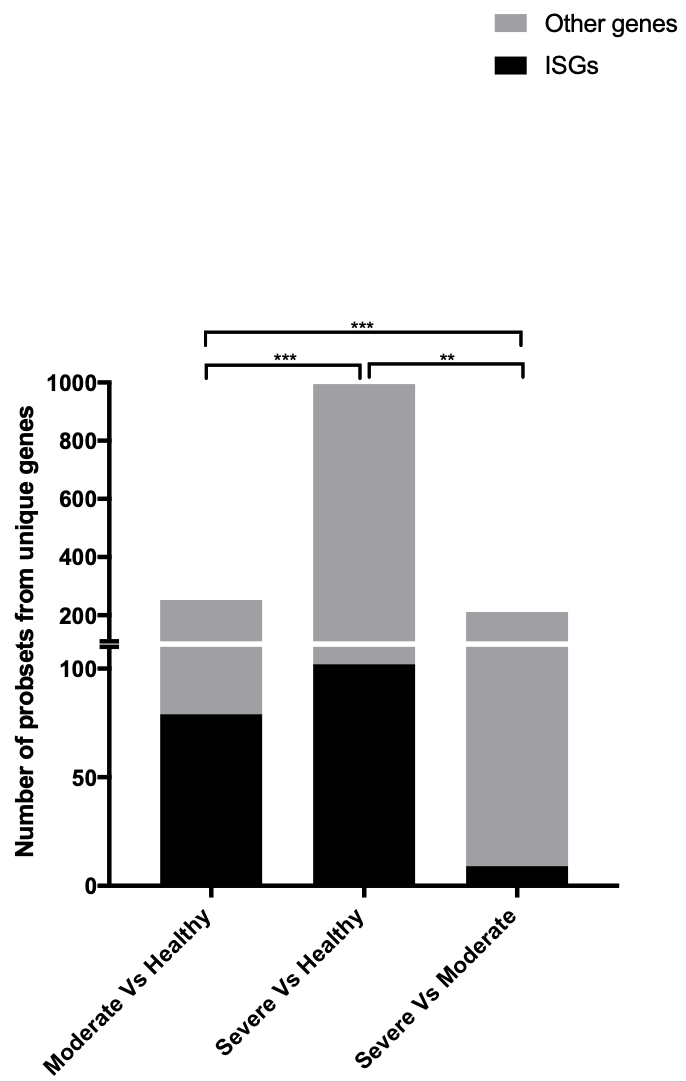
**

**Additional Figure 2**

**Number of differentially expressed genes and proportion of interferon-stimulated gene for the three comparisons.**

Y axis shows the number of differentially expressed genes.

** indicate *p value* < 0.002; *** indicate *p value* < 0,001; p values are calculated using Chi square test. ISG denotes Interferon Stimulated Gene

**Additional Figure 3**

**
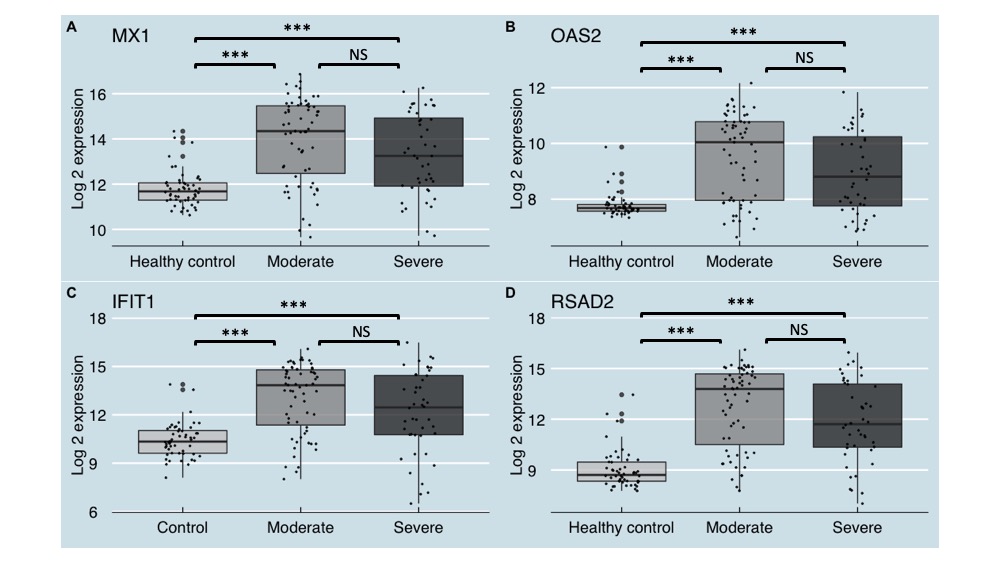
**

**Additional Figure 3: Boxplots of Interferon-stimulated genes-expression.** A: *MX1*, B: *OAS2*, C: *IFIT1*, D: *RSDA2*. Y-axis shows normalised log_2_ expression levels. Box center line: median, box limits: upper and lower quartiles. *** indicate p<0.001, adjusted for multiple testing by Bonferroni method.

ISGs were upregulated in patients with influenza illness compared to healthy control subjects. Level of expression were not significantly different between severe and moderate.

**ADDITIONAL TABLES**

**Additional table 1**

**Additional table 2**

**Additional table 3**

**Additional table 4**

**Additional table 5**

**Additional table 1**

| Pathway | Significance  -log(pValue) | Gene enrichement in pathways | FDR | Network Objects from Active Data |
| --- | --- | --- | --- | --- |
| IFN-alpha/beta signaling via JAK/STAT | 17,25 | 16/64 | 3,312E-15 | ISG54, IRF7, RSAD2, PKR, IFI17, IL1RN, XAF1, IFI27, OAS1, IFI6, Apo-2L(TNFSF10), RIG-G, PML, USP18, GBP1, ISG15 |
| NETosis | 8,95 | 8/31 | 3,269E-07 | Histone H2, Histone H2A, Leukocyte elastase, Histone H4, CAMP, Histone H1, PERM, Alpha-defensin |
| IFN-alpha/beta signaling via MAPKs | 7,96 | 10/77 | 2,131E-06 | ISG54, IRF7, RSAD2, PKR, MEK6(MAP2K6), Apo-2L(TNFSF10), RIG-G, PML, PL scramblase 1, ISG15 |
| Neutrophil-derived granule proteins | 6,01 | 7/49 | 1,438E-04 | CHI3L1, MMP-9, Leukocyte elastase, Lactoferrin, RNS2, PERM, Alpha-defensin |
| IFN-alpha/beta signaling via PI3K and NF-kB pathways | 5,06 | 8/94 | 1,033E-03 | ISG54, IRF7, RSAD2, IFI17, IFIT1, Apo-2L(TNFSF10), GBP1, ISG15 |
| Antiviral actions of Interferons | 4,67 | 6/52 | 2,097E-03 | PKR, OAS2, OAS1, 2'-5'-oligoadenylate synthetase, MxA, OAS3 |
| Neutrophils granule protein release | 4,57 | 6/54 | 2,240E-03 | CCR3, Leukocyte elastase, Lactoferrin, RNS2, CAMP, Cathepsin G |
| CD8+ T Cell recruitment and cytoxicity | 3,94 | 5/44 | 8,501E-03 | MMP-9, CD8, Leukocyte elastase, CXCR3, IL-18R1 |
| Lectin induced complement pathway | 3,67 | 5/50 | 1,255E-02 | C2, C1 inhibitor, C2b, C2a, Clusterin |
| Innate immune response to RNA viral infection | 3,63 | 4/28 | 1,255E-02 | IRF7, MDA-5, RIG-I, LGP2 |

**Additional table 1**. **Top 10 pathways ranked by statistical significance** - moderate influenza patients compared to healthy controls**.** The fraction in the third column “Gene enrichment in pathways” denote the number of genes identified in each pathway (numerator) and the total number of known genes in the pathway (denominator)

**Additional table 2**

| Pathway | Significance  -log(pValue) | Gene enrichement in pathways | FDR | Network Objects from Active Data |
| --- | --- | --- | --- | --- |
| IFN-alpha/beta signaling via JAK/STAT | 11.24 | 19/64 | 6,951E-09 | IRF7, IFI17, OAS1, GBP1, PIG3, RSAD2, IL1RN, IFI27, Apo-2L(TNFSF10), ISG15, STAT4, PKR, IFI6, ISG54, XAF1, SOCS3, Bcl-2, RIG-G, USP18 |
| CD4 T cell co-stimulation | 9.21 | 15/49 | 3,769E-07 | CXCR5, CD28, HLA-DRB1, MHC class II, HLA-DQB1, Lck, STAT4, ICOS, Bcl-6, ZAP70, CD3, HLA-DQA1, T-bet, PD-L1, CD40L(TNFSF5) |
| NETosis | 8.89 | 12/31 | 6,145E-07 | Leukocyte elastase, PKC, PAD4, Alpha-defensin, Histone H2, Histone H2A, p38 MAPK, Histone H1.2, Histone H4, CAMP, Histone H1, PERM |
| CD8+ T cell recruitment and cytotoxicity | 7.85 | 13/44 | 4,327E-06 | Leukocyte elastase, IL-18R1, CCR5, CD8, STAT4, KLRK1 (NKG2D), Antileukoproteinase 1, T-bet, MMP-9, CX3CR1, Perforin, CXCR3, Bcl-2 |
| Regulation of granulocyte development | 7.49 | 11/32 | 7,754E-06 | MAD, Leukocyte elastase, Lactoferrin, LRG, Myeloblastin, PKC, E2F1, SOCS3, C/EBPepsilon, PERM, AML1 (RUNX1) |
| Antigen Presentation | 7.24 | 12/41 | 1,178E-05 | MHC class II alpha chain, HLA-DRB1, HLA-DRB3, MHC class II, HLA-DRA1, HLA-DRB4, HLA-DRB5, CCR7, MHC class II beta chain, HLA-DRB, p38 MAPK, MEK6(MAP2K6) |
| Antigen Presentation | 7.11 | 13/50 | 1,346E-05 | CD28, Fc gamma RI, HLA-DRB1, MHC class II, MMP-8, HLA-DRB4, MHC class II beta chain, HLA-DRB, MMP-9, CD2, TLR4, CD40L(TNFSF5), BAFF(TNFSF13B) |
| CD8+ T cell recruitment | 6.40 | 12/48 | 5,981E-05 | CD8 alpha, CCL5, Fc gamma RI, MHC class II, CCR5, ITGA4, CD8, CMKLR1, CD3, P-selectin, TLR4, CX3CR1 |
| Neutrophil-derived granule proteins | 6.30 | 12/49 | 6,078E-05 | CHI3L1, Oncostatin M, Leukocyte elastase, Lactoferrin, RNS2, MMP-8, PKC, LTBR1, Fc epsilon RI gamma, Alpha-defensin, MMP-9, PERM |
| NK cells cytotoxicity | 6.28 | 10/33 | 6,078E-05 | CIITA, CCL5, MHC class II, CCR5, FasL(TNFSF6), NCAM1, KLRK1 (NKG2D), NKG2A, Perforin, CXCR3 |

**Additional table 2**. **Top 10 pathways ranked by statistical significance -** severe influenza patients compare to healthy controls. The fraction in the third column “Gene enrichment in pathways” denote the number of genes identified in each pathway (numerator) and the total number of known genes in the pathway (denominator)

**Additional table 3**

| Pathway | Significance  -log(pValue) | Gene enrichement in pathways | FDR | Network Objects from Active Data |
| --- | --- | --- | --- | --- |
| CMH II / Ag presentation | 9,96 | 9/41 | 5,442E-08 | MHC class II alpha chain, HLA-DRB5, HLA-DRA1, MHC class II beta chain, HLA-DRB1, HLA-DRB4, HLA-DRB3, MHC class II, HLA-DRB |
| NETosis | 7,94 | 7/31 | 2,858E-06 | Leukocyte elastase, Histone H2, Histone H2A, Histone H1.2, Histone H1, PERM, Alpha-defensin |
| Neutrophils differentiation | 6,53 | 6/30 | 3,658E-05 | Lactoferrin, Myeloblastin, NGAL, PERM, SOCS3, C/EBPepsilon |
| Neutrophils migration | 6,49 | 7/49 | 3,658E-05 | Leukocyte elastase, Lactoferrin, MMP-9, MMP-8, RNS2, PERM, Alpha-defensin |
| CMH II / Ag presentation | 6,43 | 7/50 | 3,658E-05 | MMP-9, MHC class II beta chain, HLA-DRB1, MMP-8, HLA-DRB4, MHC class II, HLA-DRB |
| Neutrophils differentiation | 6,36 | 6/32 | 3,658E-05 | Leukocyte elastase, Lactoferrin, Myeloblastin, PERM, SOCS3, C/EBPepsilon |
| CD8+ T cell recruitment | 4,27 | 5/44 | 3,829E-03 | Leukocyte elastase, MMP-9, CX3CR1, IL-18R1, Antileukoproteinase 1 |
| IL-5 signaling via JAK/STAT | 3,76 | 5/56 | 1,078E-02 | Survivin, IgJ, RNS2, CD24, Bcl-6 |
| IL-11 signaling via JAK/STAT | 3,56 | 4/34 | 1,517E-02 | Leukocyte elastase, Survivin, Myeloblastin, SOCS3 |
| IL-6 signaling / cell cycle | 3,42 | 4/37 | 1,903E-02 | TFF3, Survivin, Cyclin B, SOCS3 |

**Additional table 3**. **Top 10 pathways ranked by statistical significance -** severe influenza patients compare to moderate influenza patients. The fraction in the third column “Gene enrichment in pathways” denote the number of genes identified in each pathway (numerator) and the total number of known genes in the pathway (denominator)

**Additional table 4**

**
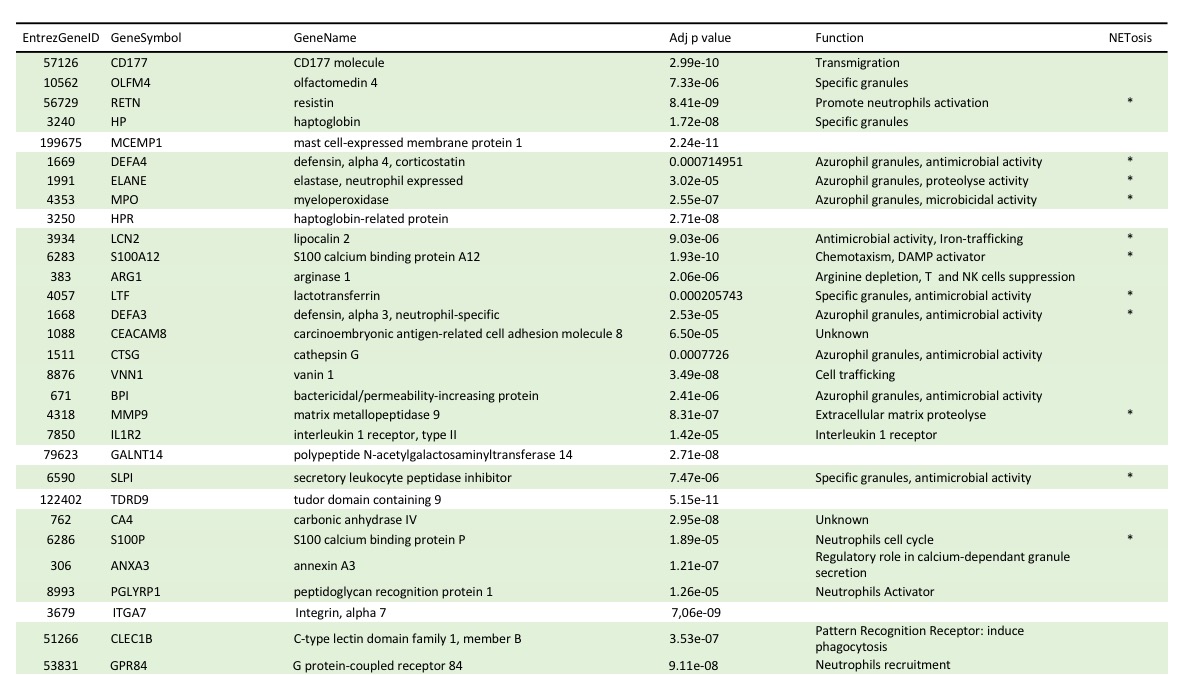
**

**Additional table 4**: **Top 30 differentially expressed genes** (ranked by expression levels) between moderate and severe infections. *p*-values were corrected for multiple testing by Benjamin/Hochberg method (“adj.P.Val”). Neutrophil-related genes are coloured.

*: involved in NETosis

: neutrophil related gene

| UP-REGULATED | | | | | |
| --- | --- | --- | --- | --- | --- |
| Our dataset | | | External dataset | | |
| Gene Symbol | logFC | adj p value | Gene Symbol | logFC | adj p value |
| **CD177** | **3.543478** | **2.985908e-10** | **OLFM4** | **3.239212** | **2.680979e-07** |
| **OLFM4** | **2.430397** | **7.331596e-06** | **ELANE** | **3.209225** | **1.404573e-08** |
| **RETN** | **2.322493** | **8.405857e-09** | LOC653600 | 3.202368 | 6.463754e-07 |
| **HP** | **2.225845** | **1.727167e-08** | **CD177** | **3.177416** | **6.620496e-11** |
| MCEMP1 | 2.167391 | 2.235318e-11 | MMP8 | 3.118558 | 1.785336e-11 |
| **DEFA4** | **1.988821** | **7.149508e-04** | **DEFA4** | **3.089101** | **2.899337e-07** |
| **ELANE** | **1.984567** | **3.021106e-05** | ZDHHC19 | 3.039518 | 1.028618e-09 |
| **MPO** | **1.982863** | **2.552539e-07** | **RETN** | **2.853591** | **4.473511e-12** |
| HPR | 1.980384 | 2.711527e-08 | **MPO** | **2.850870** | **7.155830e-10** |
| **LCN2** | **1.938694** | **9.034059e-06** | **HP** | **2.838061** | **1.888192e-13** |
| S100A12 | 1.892086 | 1.925891e-10 | **BPI** | **2.807194** | **6.434272e-11** |
| **ARG1** | **1.879958** | **2.057782e-06** | CEACAM6 | 2.795199 | 1.719735e-07 |
| **LTF** | **1.865859** | **2.057434e-04** | **CTSG** | **2.703279** | **8.260260e-07** |
| DEFA3 | 1.863514 | 2.529543e-05 | **CEACAM8** | **2.701931** | **2.322900e-07** |
| **CEACAM8** | **1.859738** | **6.498562e-05** | DEFA1B | 2.685923 | 9.009295e-07 |
| **CTSG** | **1.855233** | **7.726060e-04** | **LCN2** | **2.544993** | **4.366805e-09** |
| VNN1 | 1.761042 | 3.488464e-08 | PGLYRP1 | 2.523121 | 1.542752e-09 |
| **BPI** | **1.746079** | **2.414232e-06** | **LTF** | **2.502464** | **1.983306e-07** |
| MMP9 | 1.684753 | 8.316059e-07 | **ARG1** | **2.501436** | **1.032596e-07** |
| **IL1R2** | **1.652723** | **1.422445e-05** | **IL1R2** | **2.448910** | **2.695279e-07** |

**Additional table 5**

A.

B.

| DOWN-REGULATED | | | | | |
| --- | --- | --- | --- | --- | --- |
| Our dataset | | | Our dataset | | |
| Gene Symbol | logFC | adj p value | Gene Symbol | logFC | adj p value |
| CDKN1C | -2.290735 | 1.003602e-08 | KLRD1 | -2.020999 | 2.604969e-11 |
| LGALS2 | -2.196208 | 2.385464e-07 | FGFBP2 | -2.017702 | 7.329865e-10 |
| CSF1R | -2.169384 | 3.384837e-11 | FCER1A | -1.976502 | 2.104961e-06 |
| TGFBI | -2.130399 | 2.235318e-11 | **IFIT3** | **-1.967009** | **1.083546e-04** |
| PRKAR2A-AS1 | -1.892920 | 3.770897e-05 | **GPR56** | **-1.962116** | **5.582484e-10** |
| ARHGEF10L | -1.818692 | 2.873439e-12 | **CX3CR1** | **-1.938368** | **3.248679e-10** |
| **CX3CR1** | **-1.656473** | **4.261208e-08** | GZMH | -1.932696 | 1.319863e-08 |
| MS4A7 | -1.608028 | 1.299725e-09 | S1PR5 | -1.813299 | 7.604443e-11 |
| ZNF83 | -1.575731 | 4.426601e-05 | **IFIT1** | **-1.767648** | **7.102383e-04** |
| GLUD1P7 | -1.545367 | 1.156848e-04 | LOC649143 | -1.761794 | 1.062396e-06 |
| COL11A2 | -1.511588 | 3.343425e-04 | EOMES | -1.757606 | 3.475999e-08 |
| ZNF337-AS1 | -1.499143 | 2.116723e-04 | GNLY | -1.754399 | 3.049948e-07 |
| C21orf58 | -1.495579 | 2.062743e-04 | **IFI44L** | **-1.751042** | **3.220606e-03** |
| **GPR56** | **-1.487178** | **2.553711e-06** | **HLA-DPA1** | **-1.749806** | **2.133317e-09** |
| **HLA-DPA1** | **-1.478938** | **7.625097e-11** | HES4 | -1.733206 | 1.778969e-05 |
| MPEG1 | -1.475354 | 2.235318e-11 | **ISG15** | **-1.637426** | **1.052773e-03** |
| HLA-DPB1 | -1.474814 | 1.542643e-09 | HLA-DRB6 | -1.635289 | 4.027141e-09 |
| ARFRP1 | -1.469774 | 1.644243e-03 | KLRF1 | -1.635238 | 1.997896e-09 |
| FAM181A | -1.447345 | 8.879842e-05 | MT2A | -1.633937 | 4.613127e-05 |
| GPBAR1 | -1.433340 | 1.651632e-09 | KLRG1 | -1.630755 | 1.119425e-07 |

**Additional table 5**:

**A: Top 20 most upregulated genes in both datasets**

**B. Top 20 most downregulated genes in both datasets**

Genes highlighted in **Red** are common to both datasets.
